# JAK inhibitors impair GM-CSF-mediated signaling in innate immune cells

**DOI:** 10.1186/s12865-020-00365-w

**Published:** 2020-06-15

**Authors:** Yuya Fujita, Naoki Matsuoka, Jumpei Temmoku, Makiko Furuya-Yashiro, Tomoyuki Asano, Shuzo Sato, Haruki Matsumoto, Hiroshi Watanabe, Hideko Kozuru, Hiroshi Yatsuhashi, Atsushi Kawakami, Kiyoshi Migita

**Affiliations:** 1 Department of Rheumatology, Fukushima Medical University School of Medicine, 1 Hikarigaoka, Fukushima, Fukushima 960-1295 Japan; 2 Clinical Research Center, NHO Nagasaki Medical Center, Kubara 2-1001-1 Omura, Nagasaki, 856-8562 Japan; 3 Department of Immunology and Rheumatology, Unit of Advanced Preventive Medical Sciences, Nagasaki University Graduate School of Biomedical Sciences, Sakamoto1-7-1, Nagasaki, 852-8501 Japan

**Keywords:** Baricitinib, GM-CSF, IL-1β, Janus kinases, Rheumatoid arthritis, Tofacitinib, Upadacitinib

## Abstract

**Background:**

Innate immune cells play a crucial role in the pathophysiology of rheumatoid arthritis (RA) via release of cytokines. Small-molecule inhibitors of Janus kinases (JAKi) are clinically efficacious in patients with RA. However, the isoform-specific action of each JAKi is difficult to assess, since JAKs form heterodimeric complexes with cytokine receptors. We assessed the effects of several JAKi on GM-CSF-primed human innate immune cells.

**Results:**

Treatment with JAKi (tofacitinib, baricitinib, upadacitinib) prevented GM-CSF-induced JAK2/STAT5 phosphorylation at higher concentrations (400 nM) in THP-1 cells. Whereas compared with baricitinib or upadacitinib, the inhibitory effects of tofacitinib on the GM-CSF-induced JAK2/STAT5 phosphorylation were weak at lower concentrations (≤ 100 nM). All JAKi inhibited GM-CSF-induced IL-1β production by human neutrophils. However, the inhibitory effects of baricitinib on IL-1β production were larger compared to those of tofacitinib or upadacitinib at lower concentrations (≤ 100 nM). Similarly, all JAKi inhibited GM-CSF-induced caspase-1(p20) production by human neutrophils.

**Conclusion:**

We conclude that incubation with JAKi prevents GM-CSF-mediated JAK2/STAT5 activation in human innate immune cells. Although baricitinib and upadacitinib almost completely blocked GM-CSF-mediated JAK2/STAT5 signaling, the inhibitory effects of tofacitinib were weaker at lower concentrations suggesting that variation exists among these JAKi in the inhibition of JAK2 signaling pathways.

## Background

Cytokines play an important role in the induction of autoimmune diseases such as rheumatoid arthritis (RA) [1]. Type I/II cytokines transduce signals via the Janus kinase (JAK) / signal transduction activator of transcription (STAT) pathways [2]. Due to their important functions in cytokine signaling, JAKs have been attractive therapeutic targets in inflammatory disorders [3]. Members of JAK family are non-receptor tyrosine kinases comprised of four isoforms: JAK1, JAK2, JAK3 and TYK2 [4]. Ligation of cytokine receptors results in tyrosine phosphorylation and activation of receptor-associated JAKs and cytoplasmic STAT transcription factor, which translocate to the nucleus [5]. Therefore, inhibition of JAKs results in direct suppression of cytokine signaling pathway.

Oral JAK inhibitors (JAKi) are currently in development and have been used for treatment of RA [6]. JAKi are presumed to inhibit JAK isoforms with different selectivity [7], however, the JAK isoform selectivity of each JAKi is relative and not absolute. For example, tofacitinib was originally developed as a JAK3 inhibitor, but subsequent selectivity studies revealed additional inhibitory effects on JAK1 [8]. The selectivity profile may differ between cellular basis assay and enzymatic assay [9]. Their precise in vivo specificities remain unclear because most cytokine receptors are equipped with two different JAKs and the complexities associated with sharing of JAKs with cytokine receptors [10]. Due to heterodimeric pairing of JAKs in certain cytokine receptor, the dominant effect of one JAK over another leads to different selectivity profiles in different cytokine pathways. Therefore, in vitro enzymatic and pharmacokinetic properties of JAKi make them unsuitable for examination of in vivo immunological effects [9]. The effects of JAKi on innate immune cell signaling have been demonstrated [11]. Therefore, determining the selectivity profiles of JAKi using human innate immune cells is necessary to fully realize their potential as anti-inflammatory agents.

GM-CSF is important in the cytokine network associated with RA [12]. GM-CSF receptor is associated with JAK2 homodimer [13], therefore, JAK2 is a primary kinase regulating GM-CSF-induced signal transduction. Here we focused on GM-CSF-mediated JAK2 signaling [14] and explored the impact of JAKi (upadacitinib, baricitinib and tofacitinib), which are approved for RA treatment in Japan [15, 16]. We compared the established JAK1/3 selective (tofacitinib), JAK1/2 selective (baricitinib) and JAK1 selective (upadacitinib) JAKi, with the goal of providing insight into JAK2-mediated inflammatory processes in GM-CSF-stimulated human innate immune cells.

## Results

### Effects of JAKi on GM-CSF-stimulated THP-1 cells

**Fig. 1:**
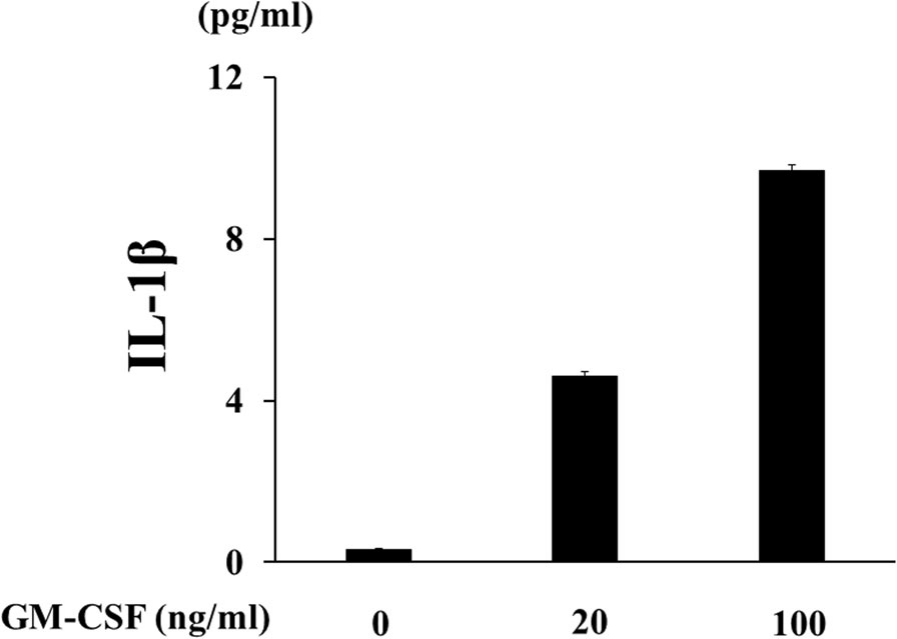
GM-CSF induces IL-1β synthesis from THP-1 cells. THP-1 cells were incubated with the indicated concentrations of GM-CSF for 24 h and supernatants were analyzed for IL-1β production by ELISA. Values represent the mean ± SD of two independent experiments

**Fig. 2:**
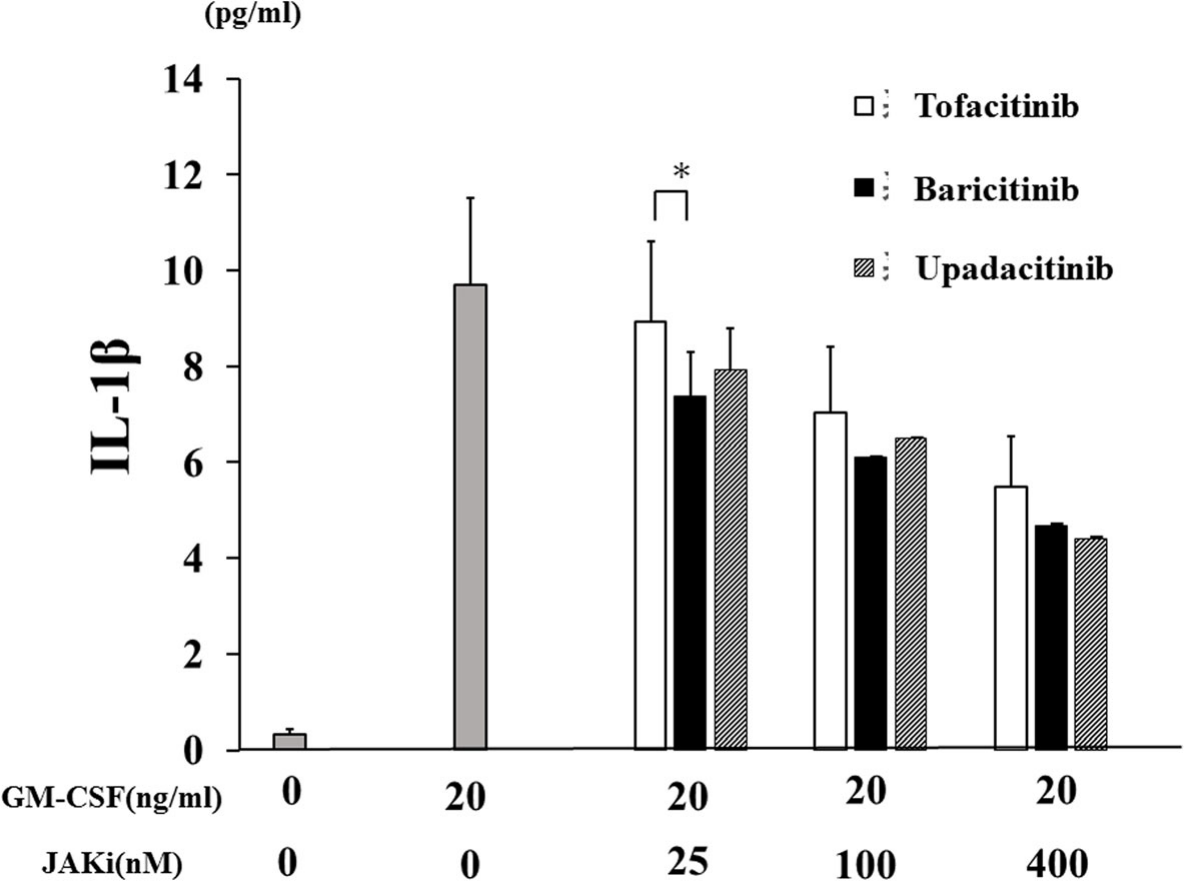
JAKi inhibit the IL-1β synthesis from GM-CSF-stimulated THP-1 cells. THP-1 cells were stimulated with GM-CSF (20 ng/ml) for 24 h in the presence or absence of the pretreatment JAKi (tofacitinib, baricitinib, upadacitinib for 1 h) and supernatants were analyzed for IL-1β production by ELISA. Values represent the mean ± SD of three independent experiments. * *p* < 0.01 baricitinib-pretreated neutrophils versus those with tofacitinib or upadacitinib

**Fig. 3:**
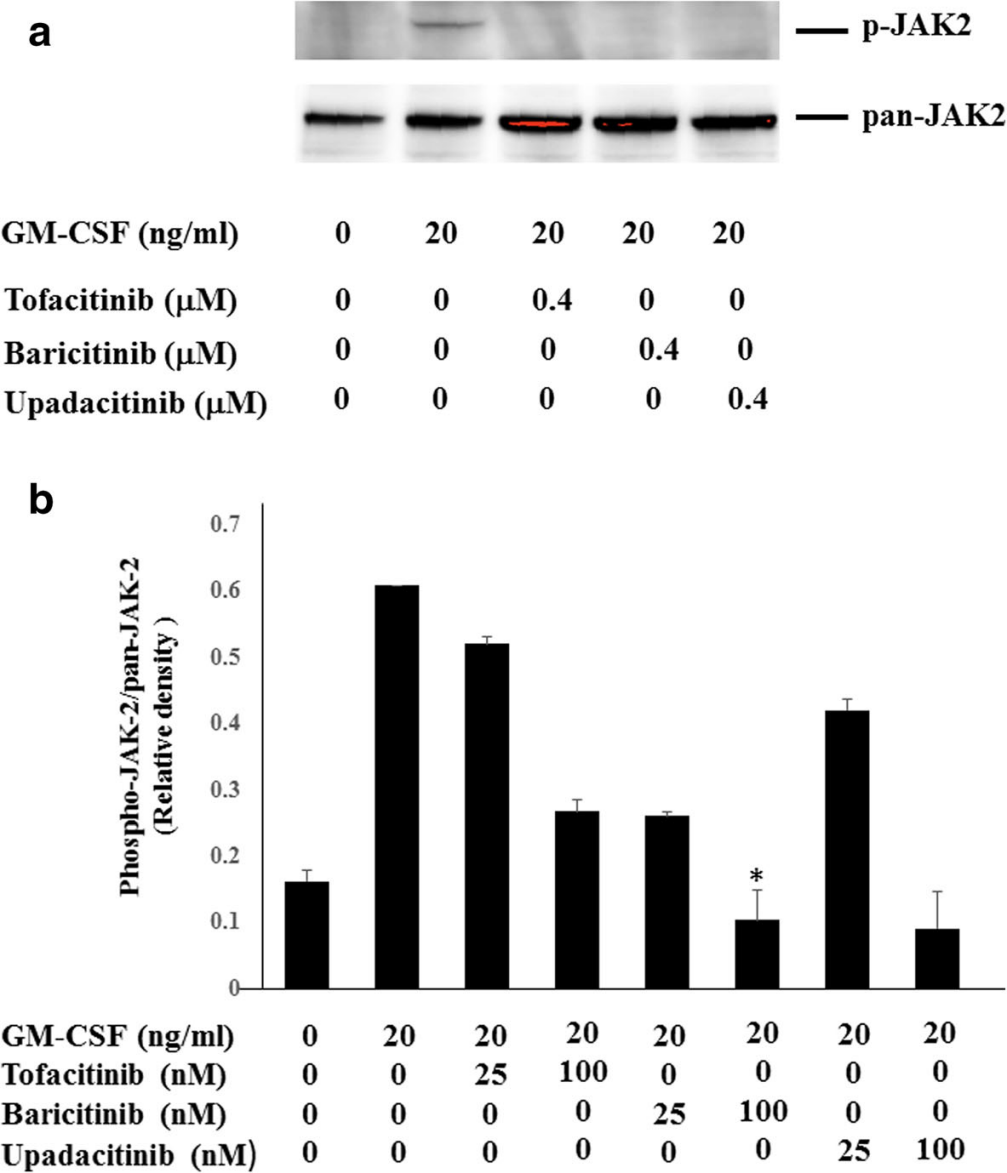
Effects of JAKi on JAK2 phosphorylation in GM-CSF stimulated THP-1 cells. **a** THP-1 cells were pretreated with JAKi (tofacitinib, baricitinib, upadacitinib) at the indicated concentrations (400 nM) for 1 h and then stimulated with GM-CSF (20 ng/ml) for 20 min. Phosphorylation of JAK2 was determined by Western blotting using phospho-specific antibodies against JAK2. **b** THP-1 cells were pretreated with JAKi (tofacitinib, baricitinib, upadacitinib) at the indicated concentrations (25, 100 nM) for 1 h and then stimulated with GM-CSF (20 ng/ml) for 20 min. Phosphorylation of JAK2 was determined by Western blotting using phospho-specific antibodies against JAK2. Phosphorylation levels of JAK2 were normalized to total protein levels. Mean ± SD of the ratio of phosphorylated JAK2 to total JAK2 of three independent experiments was shown. Statistical significance between GM-CSF-stimulated cells and each JAKi-pretreated cells was determined. *p < 0.05

**Fig. 4:**
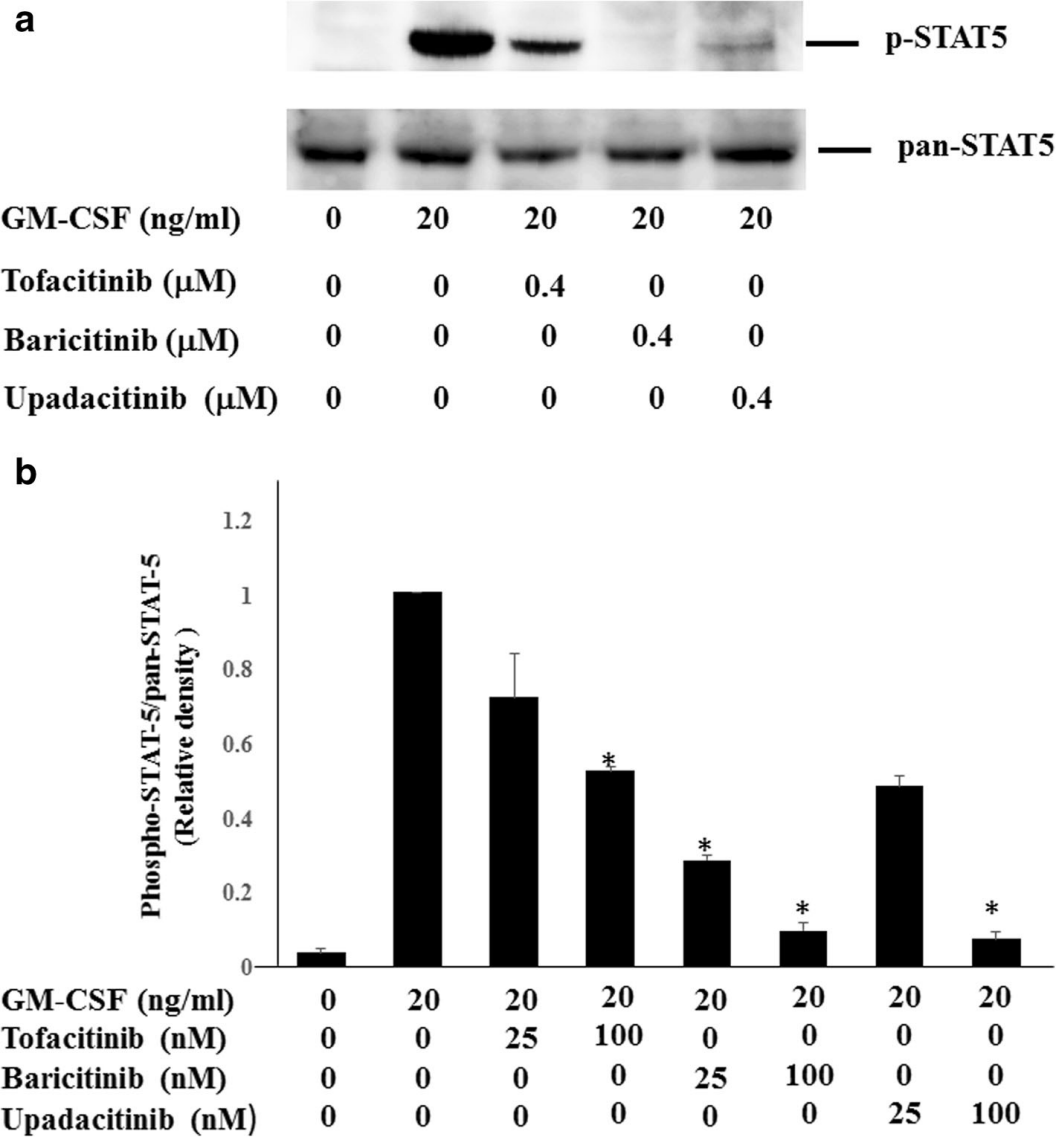
Effects of JAKi on STAT5 phosphorylation in GM-CSF stimulated THP-1 cells. **a** THP-1 cells were pretreated with JAKi (tofacitinib, baricitinib, upadacitinib) at the indicated concentrations (400 nM) for 1 h and then stimulated with GM-CSF (20 ng/ml) for 20 min. Phosphorylation of STAT5 was determined by Western blotting using phospho-specific antibodies against STAT5. **b** THP-1 cells were pretreated with JAKi (tofacitinib, baricitinib, upadacitinib) at the indicated (25, 100 nM) for 1 h and then stimulated with GM-CSF (20 ng/ml) for 20 min. Phosphorylation of STAT5 was determined by Western blotting using phospho-specific antibodies against STAT5. Phosphorylation levels of STAT5 were normalized to total protein levels. Mean ± SD of the ratio of phosphorylated STAT5 to total STAT5 of three independent experiments was shown. Statistical significance between GM-CSF-stimulated cells and each JAKi-pretreated cells was determined. *p < 0.05

To investigate whether JAKi alters cytokine production by GM-CSF-stimulated THP-1 cells, IL-1β was quantitated in culture supernatants using ELISA. We found that IL-1β production was induced by GM-CSF stimulation in THP-1 cells (Fig. 1). GM-CSF-induced IL-1β secretion by THP-1 cells was inhibited by JAKi pretreatment in a dose-dependent manner (Fig. 2). To investigate the effects of JAKi on cytokine-mediated signaling, we examined the effects of JAKi on GM-CSF-mediated JAK2/STAT activation using THP-1 cells. GM-CSF stimulation induced JAK2 phosphorylation in THP-1 cells. The results showed that the phosphorylation levels of JAK2 were decreased in JAKi-pretreated THP-I cells in dose-dependent manner, however, there were some variations. Although pretreatment with all JAKis blocked GM-CSF-induced JAK2 phosphorylation at high concentrations (400 nM, Fig. 3a), the inhibitory effects of JAKis varied at lower concentrations (Fig. 3b, Additional file 1). Baricitinib inhibited GM-CSF-induced JAK2 phosphorylation even at lower concentrations (25–100 nM). By contrast, tofacitinib weakly inhibited GM-CSF-induced JAK2 phosphorylation at the same concentrations (Fig. 3b, Additional file 1). In addition, GM-CSF stimulation induced downstream STAT5 phosphorylation, which was presumably induced by activated JAK2. However, GM-CSF stimulation barely induced STAT3 phosphorylation in THP-1 cells (data not shown). Similarly, baricitinib and upadacitinib pretreatment abolished GM-CSF-induced STAT5 phosphorylation completely at high concentrations (Fig. 4a). By contrast, the inhibitory effects of tofacitinib against GM-CSF-induced STAT5 phosphorylation were less pronounced even at high concentrations (Fig. 4a, b, Additional file 2).

### Effects of JAKi on GM-CSF-stimulated human neutrophils

**Fig. 5:**
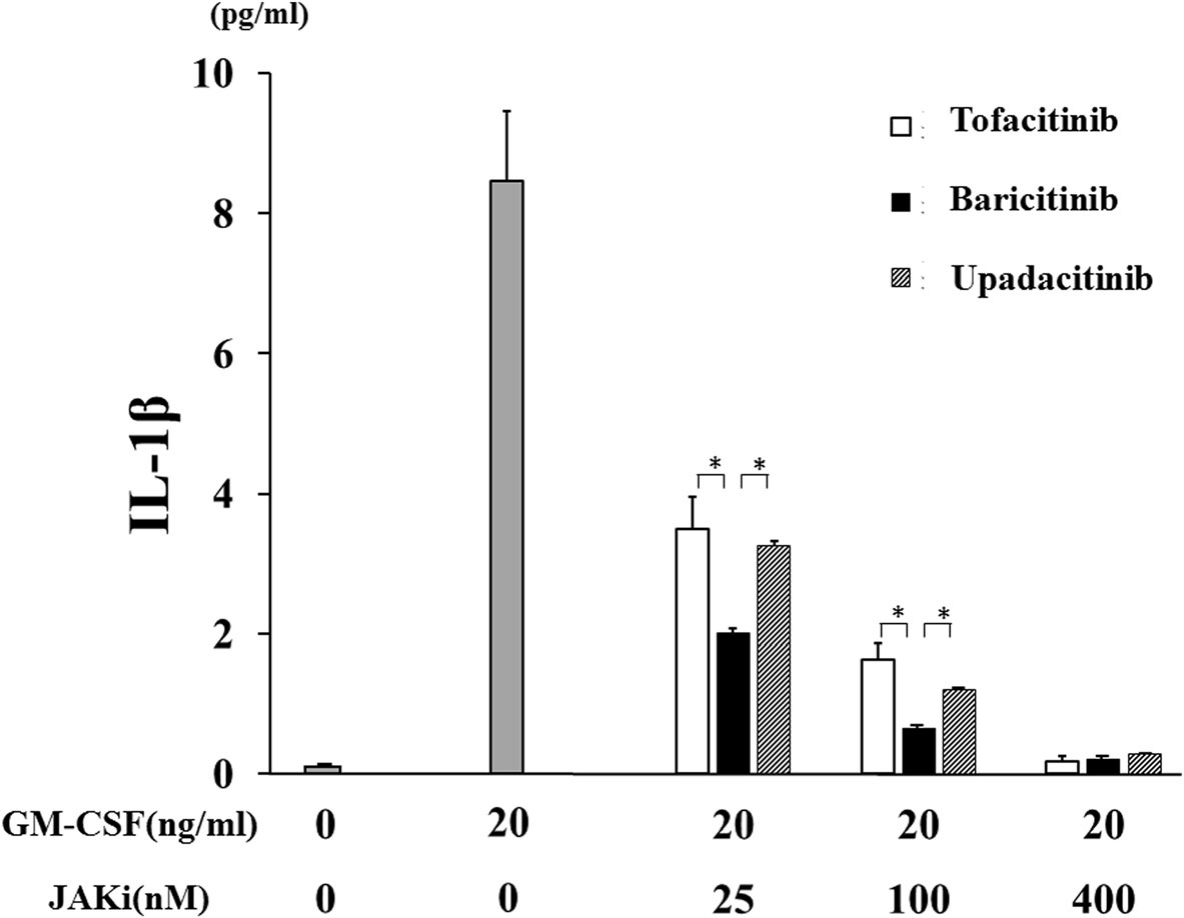
JAKi inhibit the IL-1β synthesis from GM-CSF-stimulated neutrophils. Neutrophils (1 × 10^6^/ml) were stimulated with GM-CSF (20 ng/ml) for 24 h in the presence or absence of the pretreatment JAKi (tofacitinib, baricitinib, upadacitinib for 1 h) and supernatants were analyzed for IL-1β production by ELISA . Values represent the mean ± SD of three independent experiments. * *p* < 0.01 baricitinib-pretreated neutrophils versus those with tofacitinib or upadacitinib

**Fig. 6:**
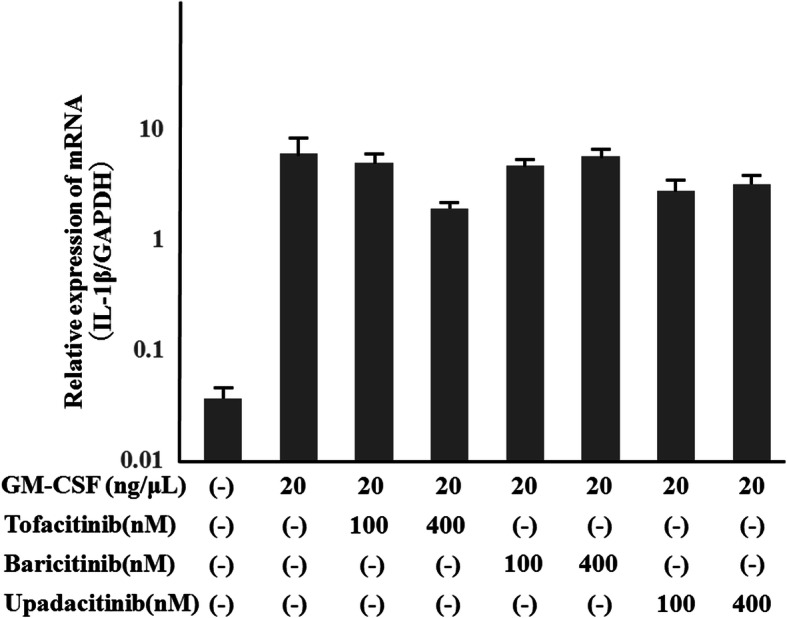
Effects of JAKi on pro-IL-1β mRNA expressions in GM-CSF-stimulated neutrophils. Neutrophils (1 × 10^6^/ml) were stimulated with GM-CSF (20 ng/ml) for 8 h in the presence or absence of the pretreatment JAKi (tofacitinib, baricitinib, Upadacitinib) for 1 h. The cells were harvested and analyzed for pro-IL-1β and GAPDH mRNA levels by real-time PCR. Values represent the mean ± SD of two independent experiments

**Fig. 7:**
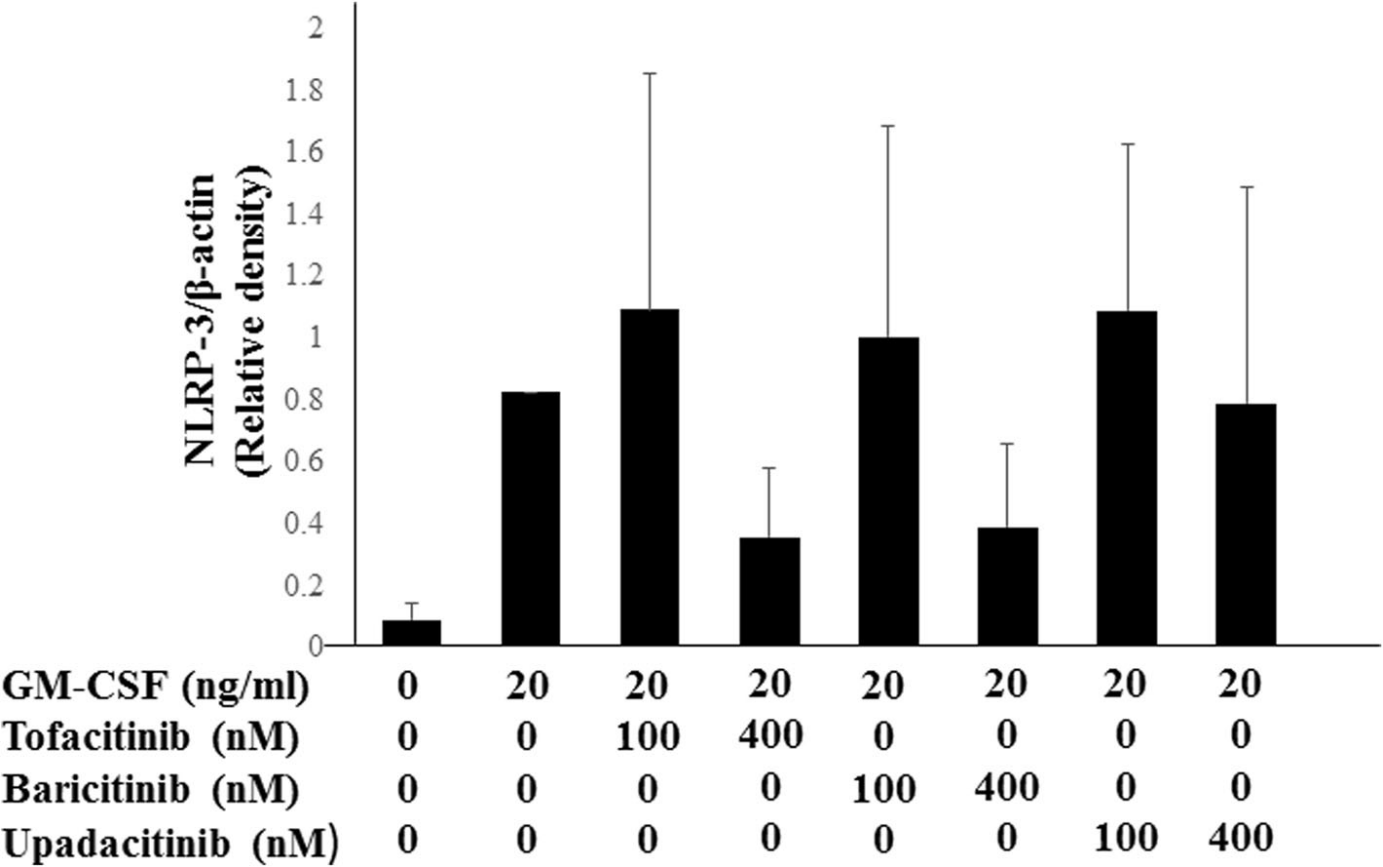
NLRP3 expression in neutrophils. Neutrophils were stimulated with GM-CSF for 24 h in the presence or absence of the pretreatment JAKi (tofacitinib, baricitinib, Upadacitinib) for 1 h. Cellular lysates were analyzed by Western using anti-NLRP3 or anti-β-actin antibodies. NLRP3 protein expression were normalized to the protein expression of β-actin. Mean ± SD of the ratio of NLRP3 to β-actin of three independent experiments was shown. Statistical significance between GM-CSF-stimulated cells and each JAKi-pretreated cells was determined. *p < 0.05

**Fig. 8:**
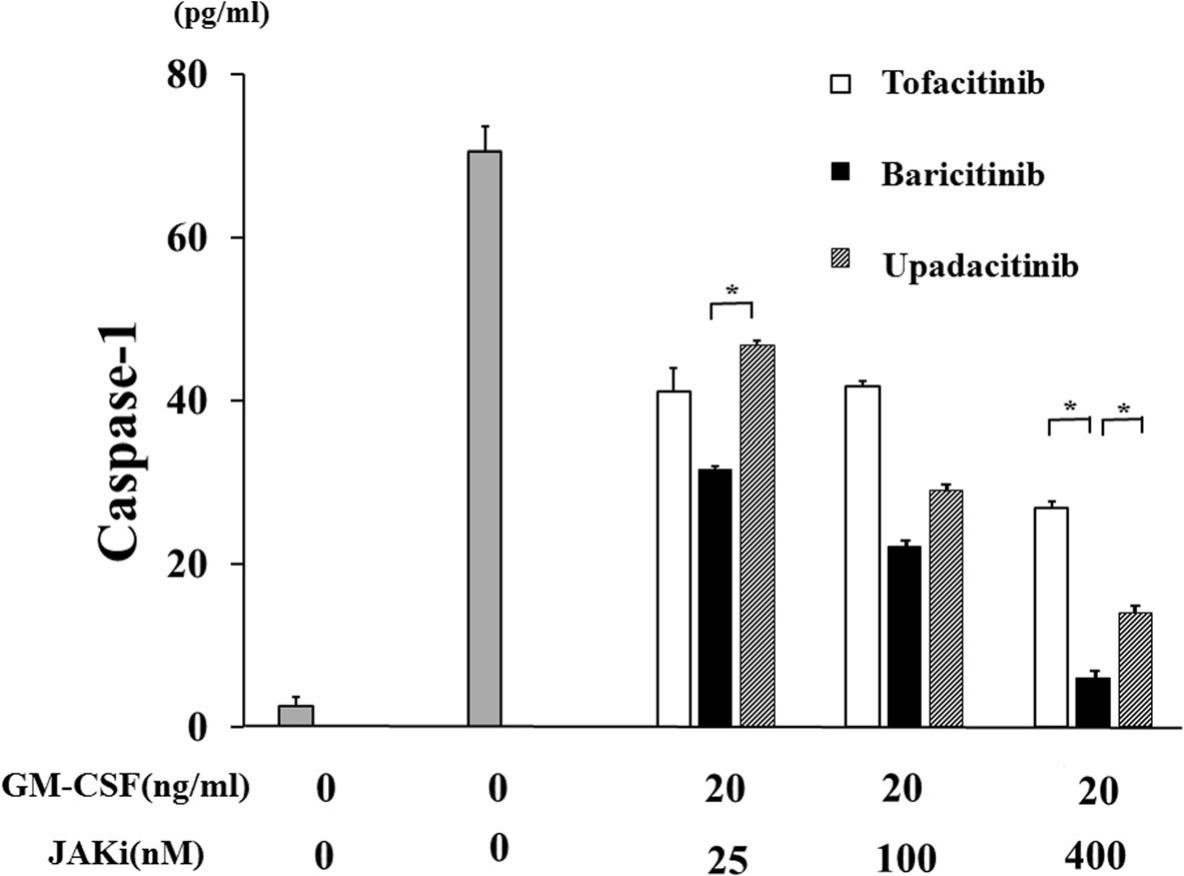
JAKi inhibit the caspase-1 (p20) release from GM-CSF-stimulated neutrophils. Neutrophils (2 × 10^6^/ml) were stimulated with GM-CSF (20 ng/ml) for 24 h in the presence or absence of the pretreatment JAKi (tofacitinib, baricitinib, upadacitinib for 1 h). Supernatants were analyzed for caspase-1 (p20) by ELISA. Values represent the mean ± SD of three independent experiments. * *p* < 0.01 baricitinib-pretreated neutrophils versus those with tofacitinib or upadacitinib

None of the JAKi studied were able to completely block IL-1β production by GM-CSF-stimulated THP-1 cells, a human monocyte-lineage tumor cell line (Fig. 2). In order to investigate the effect of each JAKi on human primary innate immune cells, we pretreated freshly isolated neutrophils with various concentrations of JAKi (tofacitinib, baricitinib, upadacitinib) for 1 h then stimulated with GM-CSF for 24 h. We assessed the production of IL-1β by GM-CSF-stimulated neutrophils. Consistent with our findings in THP-1 cells, JAKi-pretreated human neutrophils exhibited reduced GM-CSF-induced IL-1β production in a concentration-dependent manner (Fig. 5). Whereas the inhibitory effects of tofacitinib on IL-1β production by GM-CSF-stimulated neutrophils were weak in lower concentrations (25-100 nM) compared to those of baricitinib or upadacitinib (Fig. 5). We next assessed pro-IL-1β mRNA and NLRP3 protein expressions in JAKi-pretreated, GM-CSF-stimulated neutrophils. As shown in Fig. 6, GM-CSF was a potent inducer of pro-IL-1β mRNA expression in neutrophils, and JAKi pretreatment did not affect GM-CSF-induced pro-IL-1β mRNA expression. NLRP3 expression was induced in GM-CSF-stimulated neutrophils. JAKi seem to inhibit GM-CSF-induced NLRP3 expression in neutrophils, however, there was no statistical significance (Fig. 7, Additional file 3). We presented data using higher concentrations of JAKi (100–400 nM) since lower concentrations of JAKi (25 nM) had no effect on GM-CSF-induced NLRP3 expression in neutrophils (data not shown). During NLRP3 inflammasome activation, a cleaved form of caspase-1 (p20) is released along with processed IL-1β. Therefore, we analyzed culture supernatants for secretion of caspase-1 using an ELISA detecting the cleaved form of caspase-1 (p20). Consistent with IL-1β production, we found that caspase-1 secretion by GM-CSF-stimulated neutrophils was inhibited by JAKi pretreatment (Fig. 8).

## Discussion

Although there have been significant efforts to develop JAK isoform-selective inhibitors for treatment of RA, only JAK1/3 selective inhibitor (Tofacitinib) and JAK1/2 selective inhibitor (baricitinib) have been used clinically [17]. JAK2 signals in tandem with JAK1 in the heterodimeric context of gp130-family cytokine receptors such as the IL-6 receptor [18]. Therefore, it has been challenging to characterize the relative contributions of both JAK isoforms in transducing signaling from these receptors in the presence of isoform-selective JAKi. An anti-GM-CSF receptor monoclonal antibody showed similar efficacy in a clinical trial of RA patients [19]. GM-CSF may be one of the proinflammatory cytokines which contribute to the pathophysiology of rheumatoid inflammation [12]. Inhibiting JAK2 may produce an anti-inflammatory effect by inhibiting GM-CSF-mediated pathway, since GM-CSF receptor is associated with non**-**receptor protein tyrosine kinase, JAK2 [20]. We hypothesized that JAKi would differentially affect GM-CSF-mediated signaling based on their selectivity for JAK2. Our results showed that each JAKi inhibited GM-CSF-mediated JAK2/STAT5 activation and subsequent IL-1β production with some variations suggesting that the inhibitory properties of each JAKi against JAK2 could be differed.

Baricitinib, which has shown efficacy in the treatment of RA, is a potent and selective inhibitor of JAK1 and JAK2 [21]. Although tofacitinib had been developed as a JAK3-selective inhibitor, subsequent studies demonstrated that it also potently inhibits JAK1 in biochemical and cellular assays [22]. Recent clinical trials demonstrated the efficacy of upadacitinib, a selective JAK1 inhibitor, in patients with RA [23]. Biochemical assays using recombinant enzyme preparations could provide insufficient information concerning the kinase inhibition profiles of each JAKi [24]. Additionally, the pharmacological properties of JAKi demonstrated using biochemical analyses may be unsuitable for in vivo studies using human immune cells [25]. Due to the cross-inhibition of JAK isoforms forming heterodimeric complexes with various cytokines/growth factor receptors [7], inhibition assays are performed under the influence of JAK heterodimers linked to cytokine receptors in the cellular context. In this study we determined the effects of these JAKi (tofacitinib, baricitinib, upadacitinib) on key aspects of innate immune cell function. These effects are important modulators of the response to inflammatory cytokines and are implicated in an autoinflammatory reaction, IL-1β induction.

JAKi used in our study did not impair the GM-CSF-induced IL-1β mRNA expression. Synthesis of IL-1β is regulated at two separate mechanisms, one at the transcriptional activation and the other one involving translational efficiency [26]. In addition to STATs, several transcription factors including AP-1, NF-κB and C/EBPβ, activate the transcription of IL-1β gene [27–29]. Therefore, STATs inhibition may not be sufficient to regulate the IL-1β mRNA expression. On the contrary, JAKi modulated IL-1β production from innate immune cells by affecting the GM-CSF-mediated intracellular signaling pathways. This phenomenon was associated with impaired JAK2/STAT5 phosphorylation resulting in the abortive production of IL-1β without affecting the GM-CSF-induced pro-IL-1β mRNA expression in innate immune cells. These results suggest that JAKi inhibited the GM-CSF-induced inflammasome activation process, such as caspase-1 activation and subsequent IL-1β production, without affecting the inflammasome priming process [30]. Since JAK2 is associated with signaling downstream of GM-CSF [31], inhibition of JAK2 may lead to the impaired the GM-CSF-mediated autoinflammation pathway in innate immune cells [32]. We observed similarities between baricitinib and upadacitinib in the inhibitory effects on the GM-CSF-induced JAK2/STAT5 phosphorylation in innate immune cells. Similar selectivity of baricitinib and upadacitinib against JAK2 kinase may be unexpected because upadacitinib is developed as a novel JAK inhibitor engineered for JAK1 selectivity [33]. Whereas, recent study showed that upadacitinib is the most potent inhibitor among the JAKi (tofacitinib, baricitinib, upadacitinib) on the JAK2-dependent cytokines, IL-3 or GM-CSF-induced STAT5 phosphorylation in the cellular level [34]. These findings may indicate that upadacitinib would also inhibit JAK2-dependent cytokines other than those primarily dependent upon JAK1. By contrast, the suppressive effect of tofacitinib on JAK2/STAT5 phosphorylation was weaker compared to those of baricitinib or upadacitinib at lower concentrations in THP-1cells. According to previous work using enzymatic assays [35], differential effects of tofacitinib and baricitinib in inhibiting GM-CSF-induced JAK2/STAT5 phosphorylation seem to be expected. In our study, JAKi selectivity was assayed by measuring JAK/STAT phosphorylation and cytokine release using human innate immune cells. JAKi used in this study inhibited the GM-CSF-mediated JAK2/STAT5 signaling pathway, however, there were some variations. Baricitinib seems to be most potent inhibitor against GM-CSF-mediated JKA2/STAT5 pathway, followed by upadacitinib and tofacitinib. These findings also indicate that upadacitinib would inhibit JAK 2-mediated cytokine signaling.

Discrepancies may be observed in the impact and isoform selectivity of JAKi depending on the cell lineages used and cytokines measured in different assay systems [36]. Further preclinical investigations and clinical studies will be required to assess the biological impact and clinical benefit of pan-JAK or JAK isoform-selective inhibition.

There is a limitation in our study. There are some variations in GM-CSF-induced IL-1β production in THP-1 cells. Although THP-1 is an established immortalized cell line, the cells have not always exactly the same property at each experiment which may account for these variations of GM-CSF-induced cytokine productions. We utilized GM-CSF at the relatively high concentration (20 ng/ml), since these concentrations are known to be used for biochemical kinase assays [37]. Whereas the serum levels of GM-CSF in the sera from patients with RA were markedly lower [38] compared to those in our experimental conditions.

## Conclusions

We demonstrated that incubation of innate immune cells with JAKi inhibited the proinflammatory properties of GM-CSF in a dose-dependent manner. However, the inhibitory effects on JAK2/STAT5 signaling and subsequent IL-1β production were influenced by the type of JAKi. This may be due to the fine specificities of each JAKi in modulating JAK2-mediated signaling in innate immune cells. Further work focusing on the ability of each JAKi to modulate key aspects of innate immune activation and JAK2 signaling may explain the anti-inflammatory activities of JAKi for treatment of autoimmune diseases.

## Methods

### Reagents

Recombinant human GM-CSF was purchased from Peprotech (Rocky Hills, NJ). Anti-β-actin antibodies were purchased from Santa Cruz Biotechnology Inc. (Dallas, USA). Anti-NLRP-3 antibody was purchased from MERCK MILLIPORE (Billerica, MA USA). Human IL-1β and caspase-1 (p20) ELISA kits were purchased from R&D systems (Minneapolis, USA). Phospho-specific antibodies against JAK-2 (Tyr1007/1008), STAT-5 (Tyr701) and STAT-3 (Tyr705) were purchased from Cell Signaling Technology (Beverly, MA). Tofacitinib, baricinib and upadacitinib were purchased from Sigma-Aldrich (Tokyo, Japan).

### THP-1 cells

THP-1 cells were obtained from the American Type Culture Collection (Manassas, VA) and grown according to their instructions. The cells were grown in RPMI-1640 containing 10% fetal bovine serum (FBS) plus 40 U/ml penicillin, 40 μg/ml streptomycin at 37 °C, 5% CO_2_. The logarithmic growth of the cells was maintained between 2 × 10^5^ to 1 × 10^6^ cells/ml by passage, every 3–4 days. A cell concentration of 1 × 10^6^ cells /ml per well was used in a six well plate during each experiment. In the assay for JAK/STAT phosphorylation or RT-PCR, THP-1 cells were cultured with reducing amount of serum in the culture media (RPMI1640 containing 0.5% FBS) for 24 h and quiescent cells were stimulated with GM-CSF (20 ng/ml).

### Neutrophils isolation

Venous peripheral blood was collected from healthy volunteers. Written informed consent for blood donation was obtained from each individuals. The blood was layered on a Polymorphprep TM (Axis-Shield, Oslo, Norway) cushion and cells were isolated according to the manufacturer’s protocol. Briefly, neutrophils were isolated on the basis of density, washed once in 0.5 N RPMI-1640 to restore osmolality, and then washed once more in RPMI-1640. The cells were subsequently diluted in complete medium consisting of RPMI-1640.

To investigate the effects of JAKi on GM-CSF receptor signaling, freshly isolated neutrophils were pretreated with JAKi for 1 h then stimulated with GM-CSF and protein extracts or supernatants were analyzed by ELISA or immunoblotting.

### ELISA

Cell-free supernatants were collected by centrifugation at 400 g for 5 min and assayed for IL-1β or caspase-1 (p20) using ELISA kits (R&D Systems, Minneapolis, MN, USA). Quantikine human caspase-1 immunoassay (R&D Systems, Minneapolis, MN, USA) in which monoclonal antibody specific to the p20 subunit of caspase-1 was pre-coated as captured antibody and be detected by another p20-specific polyclonal antibody.

### Reverse transcription-polymerase chain reaction (RT-PCR)

Total RNA was extracted using the RNeasy total RNA isolation protocol (Qiagen, Crauley, UK) according to the manufacturer’s protocol. First-strand cDNA was synthesized from 1 μg of total cellular RNA using an RNA PCR kit (Takara Bio Inc., Otsu, Japan) with random primers. Thereafter, cDNA was amplified using specific primers respectively. The amplification of the IL-1β transcripts was also accomplished on a Light Cycler (Roche Diagnostics, Mannheim, Germany) using specific primers. The housekeeping gene fragment of glyceraldehydes-3-phosphates dehydrogenase (GAPDH) was used for verification of equal loading.

#### Cell lysis and Western blotting

Cells were stimulated with GM-CSF for the indicated times in the figure legends and the cells were washed by ice-cold PBS and lysed with RIPA Buffer (Sigma-Aldrich) supplemented with 1.0 mM sodium orthovanadate, 10 μg/mL aprotinin and 10 μg/mL leupeptin) for 20 min at 4 °C. After 5 min on ice, the cell lysates were centrifuged at 10,000 g for 10 min at 4 °C. After centrifugation, cellular lysates (30 μg) were also subjected to 12% SDS-PAGE, followed by western blot with antibodies against human NLRP3 (1:500 dilution), β-actin (1:2000 dilution) and phospho-specific anti-JAKs, and STATs antibodies (1:1000 dilution). Western blots were visualized using the enhanced chemiluminescence (ECL) system (Amersham, Little Chalfont, UK). Densitometry was done using the automated digitizing software (Image J, NIH, Bethesda, USA). All phosphorylation levels were normalized to total protein levels. NLRP3 protein expression were normalized to the protein expression of β-actin.

### Statistical analysis

Differences between groups were examined for statistical significance using Student t-test. *P* values less than 0.05 were considered statistically significance.

## Supplementary information

**Additional file 1: Figure S1.** Supplemental data for Fig. 3b. THP-1 cells were pretreated with JAKi (tofacitinib, baricitinib, upadacitinib) at the indicated concentrations (25, 100 nM) for 1 h and then stimulated with GM-CSF (20 ng/ml) for 20 min. Phosphorylation of JAK2 was determined by Western blotting using phospho-specific antibodies against JAK2. Three experiments were performed and a representative result is shown.

**Additional file 2: Figure S2.** Supplemental data for Fig. 4b. THP-1 cells were pretreated with JAKi (tofacitinib, baricitinib, upadacitinib) at the indicated (25, 100 nM) for 1 h and then stimulated with GM-CSF (20 ng/ml) for 20 min. Phosphorylation of STAT5 was determined by Western blotting using phospho-specific antibodies against STAT5. Three experiments were performed and a representative result is shown.

**Additional file 3: Figure S3.** Supplemental data for Fig. 7. Neutrophils were stimulated with GM-CSF for 24 h in the presence or absence of the pretreatment JAKi (tofacitinib, baricitinib, upadacitinib) for 1 h. Cellular lysates were analyzed by Western using anti-NLRP3 or anti-β-actin antibodies. Three experiments were performed using different neutrophils and a representative result is shown.

## Data Availability

All data generated or analysed during this study are included in this published article.

## Abbreviations

IL-1: Interleukin-1
JAK: Janus kinases
NLRP3: NLR family pyrin domain containing 3
RA: Rheumatoid arthritis
STAT: Signal transduction activator of transcription
