# Supplementary figures and images for: JAK inhibitors impair GM-CSF-mediated signaling in innate immune cells
Source: BMC Immunol. 2020 Jun 15;21:35. doi: 10.1186/s12865-020-00365-w (PMC7296727; doi:10.1186/s12865-020-00365-w)

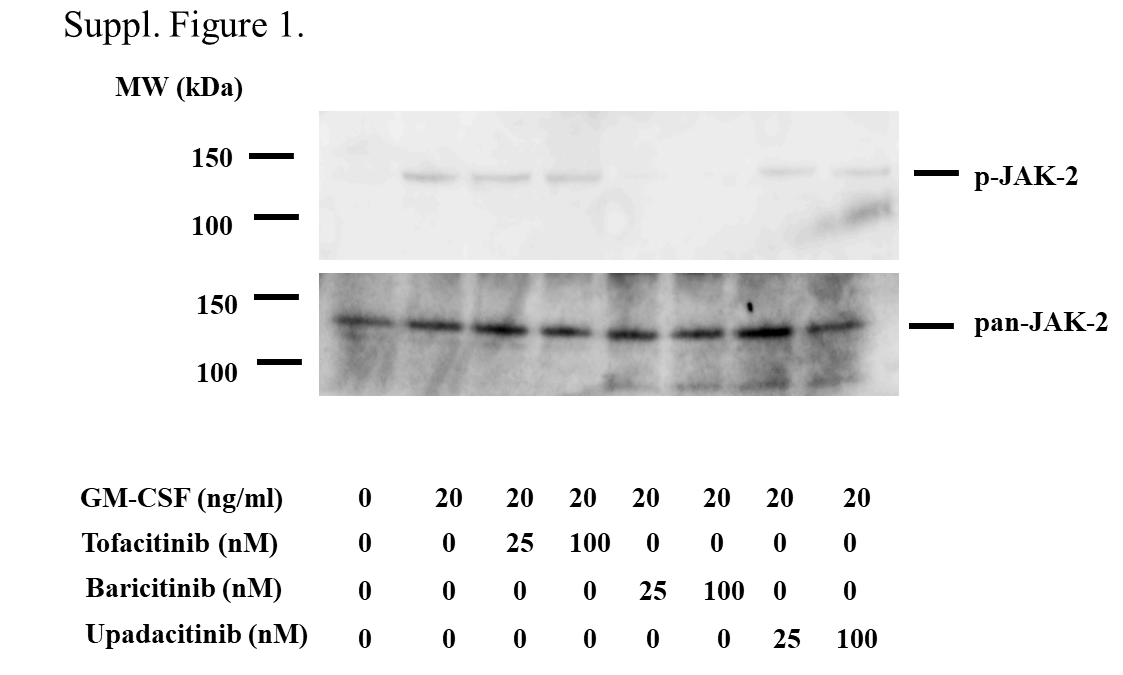

Supplement: Supplementary file 1 — Additional file 1: Figure S1. Supplemental data for Fig. 3b. THP-1 cells were pretreated with JAKi (tofacitinib, baricitinib, upadacitinib) at the indicated concentrations (25, 100 nM) for 1 h and then stimulated with GM-CSF (20 ng/ml) for 20 min. Phosphorylation of JAK2 was determined by Western blotting using phospho-specific antibodies against JAK2. Three experiments were performed and a representative result is shown. [file 12865_2020_365_MOESM1_ESM.tif]

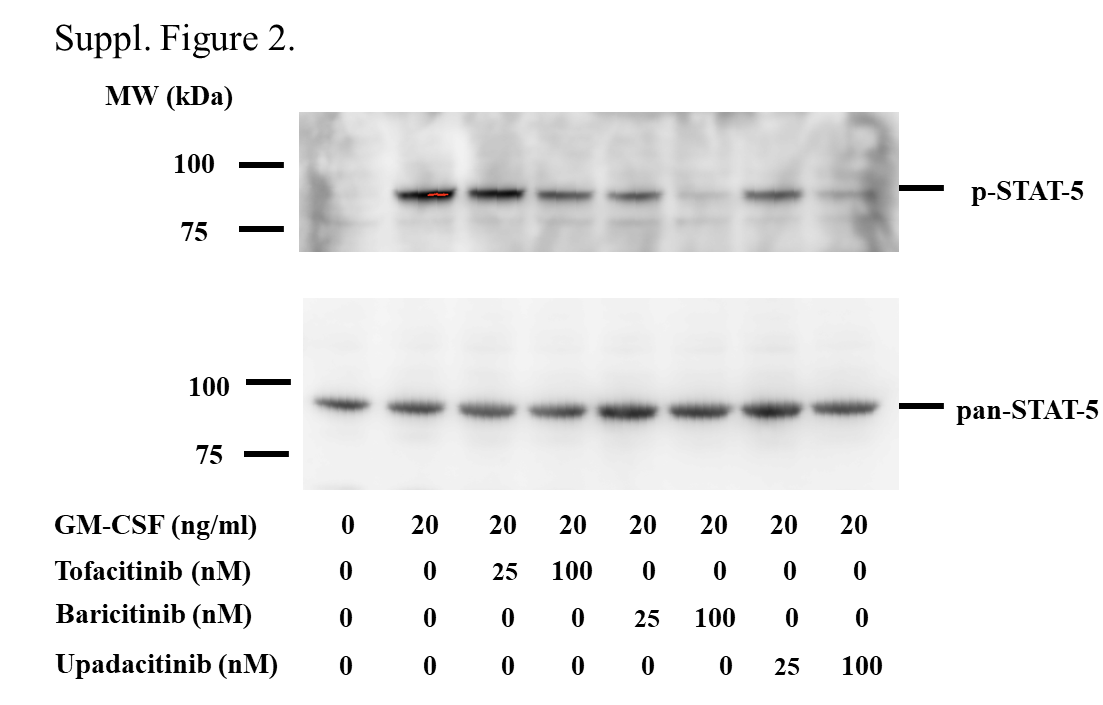

Supplement: Supplementary file 2 — Additional file 2: Figure S2. Supplemental data for Fig. 4b. THP-1 cells were pretreated with JAKi (tofacitinib, baricitinib, upadacitinib) at the indicated (25, 100 nM) for 1 h and then stimulated with GM-CSF (20 ng/ml) for 20 min. Phosphorylation of STAT5 was determined by Western blotting using phospho-specific antibodies against STAT5. Three experiments were performed and a representative result is shown. [file 12865_2020_365_MOESM2_ESM.tif]

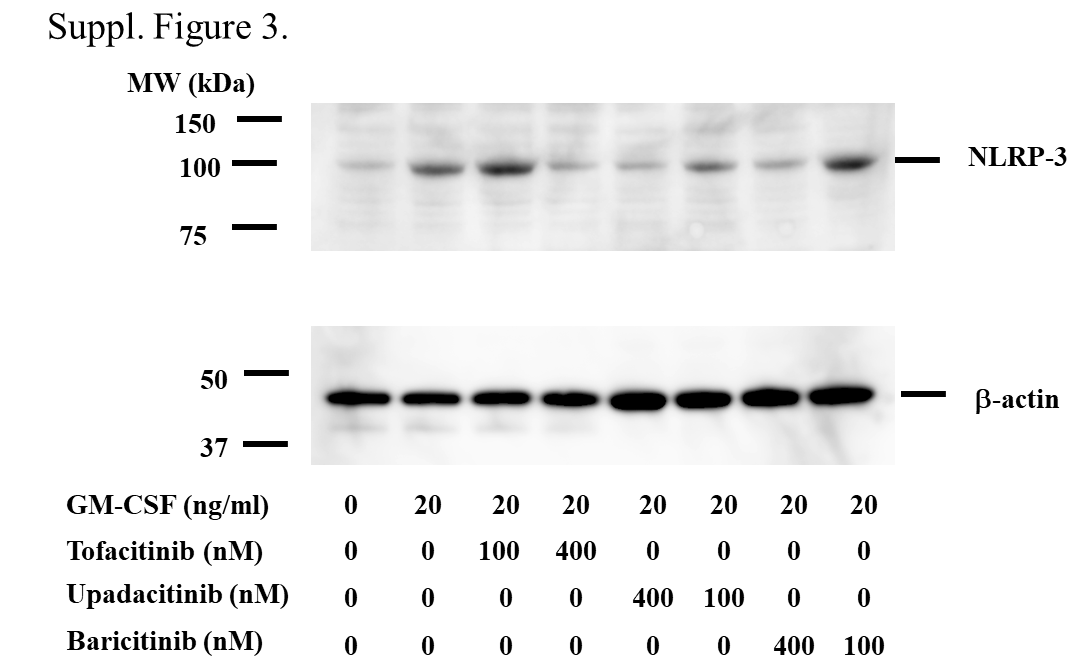

Supplement: Supplementary file 3 — Additional file 3: Figure S3. Supplemental data for Fig. 7. Neutrophils were stimulated with GM-CSF for 24 h in the presence or absence of the pretreatment JAKi (tofacitinib, baricitinib, upadacitinib) for 1 h. Cellular lysates were analyzed by Western using anti-NLRP3 or anti-β-actin antibodies. Three experiments were performed using different neutrophils and a representative result is shown. [file 12865_2020_365_MOESM3_ESM.tif]
